# Visceral obesity is associated with increased soluble CD163 concentration in men with type 2 diabetes mellitus

**DOI:** 10.1530/EC-14-0107

**Published:** 2015-01-24

**Authors:** Lars Peter Sørensen, Tina Parkner, Esben Søndergaard, Bo Martin Bibby, Holger Jon Møller, Søren Nielsen

**Affiliations:** 1 Department of Endocrinology and Internal Medicine, Aarhus University Hospital, Nørrebrogade 448000, Aarhus C, Denmark; 2 Department of Clinical Biochemistry, Horsens County Hospital, Horsens, Denmark; 3 Department of Biostatistics, Aarhus University, Aarhus, Denmark; 4 Department of Clinical Biochemistry, Aarhus University Hospital, Aarhus, Denmark

**Keywords:** soluble CD163, macrophages, inflammation, white adipose tissue, obesity, type 2 diabetes mellitus

## Abstract

Monocyte/macrophage-specific soluble CD163 (sCD163) concentration is associated with insulin resistance and increases with deteriorating glycemic control independently of BMI. This led to the proposal of the hypothesis that obesity-associated white adipose tissue inflammation varies between individuals. The objective was to examine the effect of male overweight/obesity and type 2 diabetes mellitus (T2DM) on associations between adiposity parameters and sCD163. A total of 23 overweight/obese non-diabetic men, 16 overweight/obese men with T2DM, and a control group of 20 normal-weight healthy men were included. Body composition and regional body fat distribution were determined by whole-body dual X-ray absorptiometry scan and abdominal computed tomography (CT) scan. Serum sCD163 concentrations were determined by ELISA. Associations between adiposity parameters and sCD163 were investigated using multiple linear regression analysis. In the normal-weight healthy men, there was no significant association between adiposity parameters and sCD163, whereas in the overweight/obese non-diabetic men, measures of general and regional adiposity were positively associated with sCD163. In the overweight/obese men with T2DM, only visceral adipose tissue (VAT) and the ratio of VAT to abdominal subcutaneous adipose tissue (SAT), a measure of relative body fat distribution between VAT and SAT depots, were positively associated with sCD163. In a multivariate analysis, including VAT, upper-body SAT, and lower-body fat, adjusted for BMI and age, VAT remained a significant predictor of sCD163 in the overweight/obese T2DM men, but not in the overweight/obese non-diabetic men. Our results indicate that VAT inflammation is exaggerated in men with T2DM, and that propensity to store excess body fat viscerally is particularly detrimental in men with T2DM.

## Introduction

Obesity is a major risk factor for the development of insulin resistance and type 2 diabetes mellitus (T2DM), but the adverse metabolic consequences of obesity vary between individuals (1). The mechanisms underlying obesity-associated insulin resistance remain unclear, but it is well established that not only the amount, but also the distribution of excess body fat is important, and that white adipose tissue (WAT) inflammation and insulin resistance are inter-related (2). Studies have demonstrated that expansion of visceral adipose tissue (VAT) rather than subcutaneous adipose tissue (SAT) depots is critical for the development of obesity-associated insulin resistance, and that, at least in overweight/obese subjects, expansion of SAT depots may even be protective (3, 4). However, absolute quantification of VAT and SAT fail to reflect the relative distribution of body fat, and recent data suggest that a high VAT/SAT ratio, a measure of relative body fat distribution between VAT and SAT depots, is a unique risk marker independent of overall obesity (5).

Early studies in rodent models of obesity and T2DM suggested that increased secretion of tumor necrosis factor alpha (TNFα) from adipose tissue induces insulin resistance relating WAT inflammation and insulin resistance (6). This relationship was strengthened by the discovery that, within WAT, inflammatory mediators such as TNFα and interleukin 6 (IL6) are predominantly derived from non-adipocytes, particularly macrophages (7, 8), and that obesity-associated WAT inflammation is characterized by an increased abundance of adipose tissue macrophages in mice (8, 9). In humans, studies in non-diabetic subjects have confirmed an association between BMI and WAT macrophage numbers and established that macrophage numbers are increased in VAT compared with SAT depots (10, 11, 12, 13). The propensity of VAT for increased inflammation and the subsequent release of inflammatory mediators may significantly contribute to insulin resistance in visceral obesity (14).

TNFα is expressed as a membrane-bound protein in macrophages and other immune cells (15) and is cleaved into a soluble form and released from the cell surface by proteolytic action of the metalloproteinase TNFα-converting enzyme (TACE) (16). Recently, it has been demonstrated that TACE is also responsible for the shedding of monocyte/macrophage-specific soluble CD163 (sCD163) (17).

Plasma sCD163 is regarded as a marker of macrophage activity and a long-circulating marker of TNFα (17), and concentrations of sCD163 are increased in obesity (18, 19, 20, 21, 22) and T2DM (18, 23, 24). In a retrospective cohort study, it has been demonstrated that baseline increased sCD163 concentration is a strong risk marker for developing T2DM in the general population independently of BMI and age (25). Furthermore, in recent cross-sectional studies, we and others have demonstrated that sCD163 is strongly associated with insulin resistance as assessed by HOMA-IR (20, 21, 24) and euglycemic hyperinsulinemic clamp technique (26), which was evident in normal-weight and obese healthy individuals (20, 21, 26), as well as in individuals with normal glucose tolerance (NGT), impaired glucose tolerance (IGT), and T2DM (24). Interestingly, in our study including subjects with NGT, IGT, and T2DM, sCD163 as well as TNFα, IL6, and CRP concentrations increased with deteriorating glycemic control even though the groups were BMI matched (BMI ∼30 kg/m^2^) (24). To the extent sCD163 concentrations reflect WAT inflammation, this is in accordance with varying obesity-associated WAT inflammation between individuals, possibly due to depot-specific differences. WAT macrophages highly express *CD163* mRNA (19), and *CD163* mRNA expression in SAT and VAT is positively associated with sCD163 (26). Consistent with this, studies in non-diabetic subjects have demonstrated that measures of adiposity including BMI, waist circumference, SAT, and VAT are positively associated with sCD163 (20, 21, 22). However, the effects of T2DM and relative body fat distribution on these associations have not been studied previously. In this study, groups of overweight/obese non-diabetic men (overweight/obese men) and overweight/obese men with T2DM (overweight/obese T2DM men) as well as a ‘control group’ of normal-weight healthy men (normal-weight men) were included to assess the effect of overweight/obesity and T2DM on the association between adiposity parameters and sCD163. The ratio of VAT to abdominal SAT (VAT/SAT) was included as a measure of relative body fat distribution.

Besides WAT macrophages, fixed hepatic macrophages (Kupffer cells) may be an important source of sCD163, and, in the context of this study, it would have been interesting to include liver fat in the analyses. Unfortunately, data on liver fat were not available, and instead we included alanine transaminase (ALAT), an indicator of hepatocellular damage, which is associated with liver fat content (27), in the analyses.

## Subjects and methods

### Subjects

This study is based on data from three previously published metabolic studies (28, 29, 30), but no data on sCD163 in these cohorts have been published. All study protocols were approved by the Local Ethics Committee and informed consent was obtained from all participants.

In the first study, 11 overweight/obese (BMI ≥25 kg/m^2^) men with T2DM and 11 BMI- and age-matched non-diabetic men were included (28). In the second study, 12 obese (BMI ≥30 kg/m^2^) non-diabetic men and 12 normal-weight (BMI <25 kg/m^2^) age-matched healthy men were included (29). In the third study, eight overweight/obese men with T2DM and eight normal-weight age-matched healthy men were included (30). Three subjects with T2DM participated in both the first and the second studies and only data from the first study were used in these cases. Thus, in total, 20 normal-weight men, 23 overweight/obese men, and 16 overweight/obese T2DM men were included. All participants were non-smokers with no history of alcohol abuse. In all groups, some of the participants were recreationally active, but none were elite trained. T2DM was diagnosed according to existing WHO diagnostic criteria. None of the non-diabetic men had first-degree relatives with known T2DM. Diabetes treatments were lifestyle modifications alone in seven patients or either metformin or sulfonylurea or both in remaining patients. Normal blood count and thyroid, liver, and kidney functions were confirmed in all participants.

### Study design

The study procedures before the actual study day were identical in the three study protocols. At a screening visit, potential participants visited the laboratory in the fasted state. The medical history was obtained, a physical examination was performed, and blood samples were drawn for screening purposes. One week before the study day, included participants visited again. Dual X-ray absorptiometry (DXA) scan and abdominal CT scan were performed to determine body composition and regional fat distribution. The participants were interviewed by a dietitian who estimated individual daily caloric intake based on which a weight-maintaining diet (55% carbohydrate, 15% protein, and 30% fat) was provided by the hospital kitchen during the 3 days preceding the study day. Oral hypoglycemic agents were discontinued 3 weeks before the study day while lipid-lowering and antihypertensive therapy was discontinued 2 weeks before the study day.

The participants were admitted on the evening before the study day. After an overnight fast, baseline blood samples were drawn. The samples were placed on ice and separated as quickly as possible by centrifugation (2,753 ***g*** at 4 °C for 10 min). Glucose concentrations were measured immediately, and serum samples were stored at −20 °C for later analysis of sCD163 and insulin. HbA1c, ALAT, triglyceride, and cholesterol concentrations were measured in the blood samples obtained at the screening visit.

### Body composition and regional body fat distribution

Total body fat and fat percentage were determined by DXA scan (QDR.2000; Hologic, Inc.). Upper-body (UB), abdominal, and lower-body (LB) fat were determined using the region of interest program (31). CT scans at the L2–L3 interspace were used to determine the ratio of VAT to abdominal SAT area. VAT was estimated by multiplying this ratio by abdominal fat (31). UB SAT and abdominal SAT were calculated by subtracting VAT from UB fat and abdominal fat respectively. VAT/SAT was calculated by dividing VAT by abdominal SAT.

### Laboratory assessments

HbA1c, ALAT, triglyceride, and cholesterol concentrations were measured using standard clinical biochemical methods. Plasma glucose concentrations were measured using an YSI 2300 STAT Plus glucose analyzer (YSI, Inc., Yellow Springs, OH, USA). Serum insulin concentrations were measured using an immunoassay kit (Dako, Inc., Glostrup, Denmark). We determined serum sCD163 concentrations in duplicate samples that had been frozen for up to 3 years at −20 °C by use of an in-house sandwich ELISA on a BEP-2000 ELISA analyser (Dade Behring, Inc., Marburg, Germany) (32). In each run of 36 samples, we co-analyzed control samples and serum calibrator with concentrations traceable to purified CD163. The interassay imprecision in the current studies (seven runs) was 3.0% coefficient of variation (CV) at a concentration of 1.28 mg/l, and 7.5% CV at 3.37 mg/l. The limit of detection was 6.25 μg/l. sCD163 is robust to thawing, and stability has been rigorously verified for at least 7 months at −20 °C (32).

### Insulin resistance

Insulin resistance was estimated from fasting plasma insulin and glucose concentrations using HOMA (33). The HOMA model, version 2, from 1996, was used (34).

### Statistical analyses

Baseline characteristics in the three groups (normal-weight men, overweight/obese men, and overweight/obese T2DM men) were compared using one-way ANOVA (normally distributed data) or the Kruskal–Wallis test (non-normally distributed data). Pairwise comparisons were performed using *t*-tests or Wilcoxon's rank sum test where appropriate. The association between adiposity parameters and ALAT vs sCD163 was investigated using a multiple linear regression model on a log–log scale. Three models were considered: a simple linear regression model just including each of the different adiposity parameters and ALAT separately vs sCD163, a multiple linear regression model (model 1) that additionally included BMI and age (except for BMI which was only adjusted for age), and a multiple linear regression model (model 2) including UB SAT, VAT, and LB fat as covariates and adjusting for BMI and age. The association between the adiposity parameters and ALAT vs sCD163 was investigated in the three groups by including interaction terms between the adiposity parameters and ALAT and group. The appropriateness of the multiple linear models to describe the data was established by inspecting plots of the standardized residuals against the fitted values and the different covariates. The data were analyzed using R version 2.15.0.

## Results

### Measures of adiposity, ALAT, and age

Measures of general (BMI, weight, total body fat, and fat percentage) and regional (UB fat, UB SAT, abdominal fat, abdominal SAT, VAT, and LB fat) adiposity were, as expected, lower in the normal-weight men compared with the overweight/obese men and the overweight/obese T2DM men, whereas VAT/SAT was comparable in all groups (*P*=0.48) (Table 1). In the overweight/obese men and the overweight/obese T2DM men, BMI, weight, total body fat, fat percentage, UB fat, UB SAT, abdominal fat, abdominal SAT, and LB fat were similar although there was a tendency toward greater fat percentage (*P*=0.06) and UB fat (*P*=0.09) in the overweight/obese T2DM men compared with the overweight/obese men, and VAT was significantly increased in the overweight/obese T2DM men (*P*=0.04). ALAT concentration was significantly lower in the normal-weight men compared with the overweight/obese men and the overweight/obese T2DM men (both *P*<0.001). In the overweight/obese men and the overweight/obese T2DM men, there was no significant difference in ALAT concentration (*P*=0.11). The three groups were not matched for age (*P*=0.001). While there was a tendency toward lower age in the normal-weight men compared with overweight/obese men (*P*=0.06), age was significantly lower in the normal-weight men compared with the overweight/obese T2DM men (*P*<0.001). Age was significantly higher in the overweight/obese T2DM men compared with the overweight/obese men (*P*=0.04).

### Soluble CD163

Median sCD163 concentrations were 1.4 mg/l (range: 0.8–1.7) in the normal-weight men, 1.5 mg/l (0.5–2.8) in the overweight/obese men, and 2.1 mg/l (0.7–3.0) in the overweight/obese T2DM men (*P*=0.009) (Table 1). While the concentrations were similar in the normal-weight men and the overweight/obese men, the concentration was significantly lower in the normal-weight men compared with the overweight/obese T2DM men (*P*=0.005). The concentration was significantly greater in the overweight/obese T2DM men compared with the overweight/obese men (*P*=0.01).

### Association between measures of adiposity and ALAT vs sCD163

**Figure 1:**
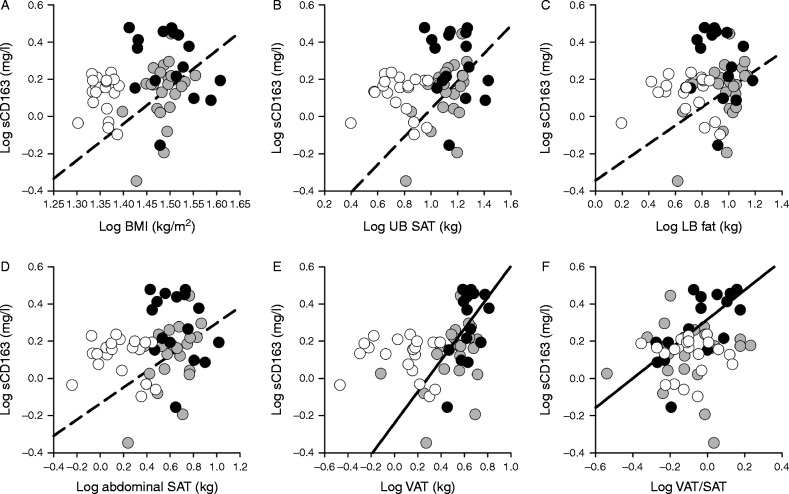
The relationship between measures of adiposity and soluble CD163 (sCD163). (A) BMI, (B) UB SAT, (C) LB fat, (D) abdominal SAT, (E) VAT, and (F) VAT/SAT. White symbols, normal-weight healthy men; grey symbols, overweight/obese non-diabetic men; black symbols, overweight/obese men with T2DM. Broken regression lines, overweight/obese non-diabetic men; solid regression lines, overweight/obese men with T2DM. Regression lines are only shown for statistically significant relationships.

In the normal-weight men, there was no significant correlation between measures of general and regional adiposity, VAT/SAT or ALAT vs sCD163 (Table 2 and Fig. 1). However, in the overweight/obese men, all these measures except VAT/SAT correlated positively with sCD163 although the correlation between VAT and sCD163 was only borderline statistically significant (*P*=0.05). In contrast, in the overweight/obese T2DM men, only VAT (*P*=0.01), VAT/SAT (*P*=0.002), and ALAT (*P*<0.0001) correlated positively with sCD163. The associations were largely unchanged after adjustment for BMI and age (Table 2).

**Figure 2:**
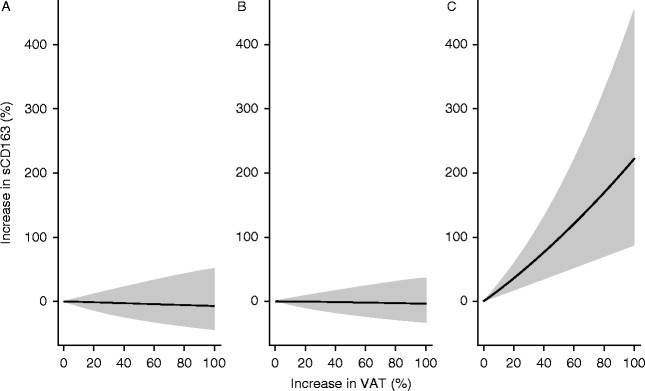
The relationship between an increase in VAT (%) and the corresponding increase in sCD163 (%) in multivariate analysis model 2 including UB SAT, VAT, and LB fat and adjusted for BMI and age. (A) Normal-weight healthy men, (B) overweight/obese non-diabetic men, and (C) overweight/obese men with type 2 diabetes mellitus. Shaded areas correspond to 95% CI.

In a multivariate analysis, including VAT, UB SAT, and LB fat, adjusted for BMI and age, VAT remained a significant predictor of sCD163 in the overweight/obese T2DM men (*P*<0.0001), but not in the overweight/obese men (*P*=NS) (Table 3 and Fig. 2).

### HOMA-IR, insulin, glucose, HbA1c, triglyceride, and cholesterol

HOMA-IR and insulin concentration were lower in the normal-weight men compared with the overweight/obese men and the overweight/obese T2DM men (both *P*<0.001), indicating greater insulin sensitivity (Table 1). However, while glucose concentration and HbA1c were comparable in the normal-weight men and the overweight/obese men, these parameters were significantly increased in the overweight/obese T2DM men (both *P*<0.001). HOMA-IR, insulin and glucose concentration, and HbA1c were greater in the overweight/obese T2DM men compared with the overweight/obese men (all *P*≤0.004). For triglyceride and cholesterol concentrations, see Table 1.

## Discussion

This study was spurred by our previous observation that sCD163, as well as TNFα, IL6, and CRP concentrations, increases with deteriorating glycemic control in BMI-matched groups of subjects with NGT, IGT, and T2DM (24), which is in accordance with obesity-associated WAT inflammation varying between individuals, possibly due to depot-specific differences. To better understand the relationship between adiposity parameters and sCD163 and the effect of overweight/obesity and T2DM on that relationship, we evaluated the association between measures of adiposity and relative body fat distribution vs sCD163 in groups of overweight/obese men, overweight/obese T2DM men, as well as a ‘control group’ of normal-weight men. In the normal-weight men, there was no significant association between adiposity parameters and sCD163, whereas in the overweight/obese men, BMI and other measures of general and regional adiposity were positively associated with sCD163. In contrast, in the overweight/obese T2DM men, only VAT and VAT/SAT were positively associated with sCD163. Moreover, the results were largely unchanged after adjustment for BMI and age.

In agreement with our previous results sCD163 concentration was significantly greater in the overweight/obese T2DM men compared with the overweight/obese men despite comparable BMI (24). However, there was no significant difference in sCD163 concentration between the overweight/obese men and the normal-weight men. This is surprising given results in previous studies (20, 21, 22) reporting greater sCD163 concentration in obese men compared with normal-weight men (22), as well as in obese men and women compared with normal-weight men and women, with no significant difference between sexes (20, 21). This difference may be explained by the inclusion of overweight/obese subjects (BMI ≥25 kg/m^2^) rather than strictly obese (BMI >30 kg/m^2^) subjects and the relatively low number of subjects in this study.

The positive associations between measures of general and regional adiposity in the overweight/obese men are, however, in agreement with results obtained from previous studies (20, 21, 22), where positive associations between sCD163 and BMI in obese and normal-weight men (22), and between sCD163 and BMI, waist circumference (21), VAT, and SAT (20) in obese and normal-weight men and women were reported. However, the lack of significant associations between adiposity parameters and sCD163 in the normal-weight men appears to be in contrast with results obtained from previous studies (20, 21, 22), but, in these studies, data on the associations between measures of adiposity and sCD163 in the sub-groups (obese and normal-weight subjects) were not reported.

The novel finding that, in the overweight/obese T2DM men, out of the included measures of general and regional adiposity, only VAT was positively associated with sCD163 suggests that VAT is an important predictor of sCD163 concentration in T2DM. In support of this, in a multivariate analysis, including VAT, UB SAT, and LB fat, adjusted for BMI and age, VAT remained a significant predictor of sCD163 in the overweight/obese T2DM men, but not in the overweight/obese men. Increased release of sCD163 from VAT may be due to increased VAT and/or exaggerated VAT inflammation. Indeed, despite comparable weight and BMI, VAT was significantly greater in the overweight/obese T2DM men compared with the overweight/obese men, which is in accordance with previous findings (27). However, at the same time, VAT/SAT, which was comparable in all groups, was positively associated with sCD163 only in the overweight/obese T2DM men, suggesting that VAT inflammation is exaggerated, and that propensity to store excess body fat viscerally is particularly detrimental in men with T2DM.

In biopsy studies conducted by Tordjman *et al*. (35) and Cancello *et al*. (36) in BMI-matched morbidly obese non-diabetic subjects and subjects with T2DM, macrophage numbers were increased in VAT compared with SAT, but with no significant difference in VAT and SAT macrophage numbers between the groups. At the same time, the VAT macrophage number was positively associated with fasting glucose, insulin, TG, liver transaminases, and histopathological components of non-alcoholic fatty liver disease (NAFLD) including steatosis and fibroinflammatory lesions, and negatively associated with HDL-cholesterol and a marker of insulin sensitivity, QUICKI. Strikingly, in both of these studies, none of these associations were found with the SAT macrophage number, and, generally, the histopathological lesions were aggravated by T2DM. The mechanisms underlying these deleterious associations might involve increased delivery of macrophage-derived inflammatory mediators and NEFA to the liver (37, 38). Still, it seems paradoxical that the VAT macrophage number is associated with deleterious metabolic and histopathological effects and that, at the same time, the VAT macrophage number is comparable in BMI-matched non-diabetic subjects and subjects with T2DM (35, 36). However, despite comparable macrophage number, VAT inflammation may still be exaggerated in terms of macrophage activity, and, in addition, data on VAT and relative body fat distribution were not provided.

Besides WAT macrophages, Kupffer cells are a potentially important source of sCD163. sCD163 concentration is highly increased in various liver diseases characterized by inflammation and fibrosis, e.g. viral hepatitis (39) and cirrhosis (40), and, in a recent study in obese children, sCD163 was increased in children with increased ALAT, steatosis, and high pediatric NAFLD fibrosis index (41). Liver fat content is closely associated with obesity, but is greater in T2DM patients compared with BMI-matched non-diabetic subjects (27). In the context of this study, it would have been interesting to include liver fat in our analyses to determine whether liver fat is also a risk marker for increased inflammation in T2DM. However, liver fat was not measured. Yet, we observed strong, positive correlations between ALAT and sCD163 in the overweight/obese men and the overweight/obese T2DM men.

Strengths of the study include the inclusion of appropriate study groups to study both the effect of overweight/obesity and T2DM, and the inclusion of VAT/SAT as a measure of relative body fat distribution. However, a group of normal-weight T2DM men were not included, and our results in the overweight/obese T2DM men may not be applicable to the subgroup of normal-weight T2DM men. The study has some weaknesses. The overweight/obese T2DM men were significantly older than the normal-weight men and the overweight/obese men. Age, however, was included in the multiple linear regression analysis and did not prove to be a significant confounder in the study. The number of study subjects is relatively low, and the results should be confirmed in larger studies. The liver represents a potentially important source of sCD163 and, in future studies dealing with the relationship between body fat distribution and sCD163, it would be appropriate to include liver fat as a predictor of sCD163. The effect of sex and ethnicity also remains to be determined.

In conclusion, our data confirm results obtained from previous studies, showing that measures of general and regional obesity are positively associated with sCD163 in overweight/obesity and that sCD163 concentration is increased in T2DM. As a novel finding, our results suggest that VAT inflammation is exaggerated in men with T2DM, and that propensity to store excess body fat in VAT, and possibly the liver, is particularly detrimental in men with T2DM. These results may add further explanation of our previous observation that sCD163 concentration increases with deteriorating glycemic control in BMI-matched groups of subjects with NGT, IGT, and T2DM, and the common observation that the adverse metabolic consequences of obesity vary between individuals.

## Author contribution statement

L P Sørensen produced data and wrote the manuscript. T Parkner wrote the manuscript. E Søndergaard produced data and reviewed/edited the manuscript. B M Bibby performed the statistical analyses and reviewed/edited the manuscript. H J Møller produced data, contributed to the discussion, and reviewed/edited the manuscript. S Nielsen designed the original metabolic studies, contributed to the discussion, and reviewed/edited the manuscript.

## Figures and Tables

**Table 1:** Characteristics of the study population. Data are expressed as mean±s.d. or median (range).

	**Group**			***P***			
Normal-weight healthy men (1)	Overweight/obese non-diabetic men (2)	Overweight/obese men with T2DM (3)	All	1 vs 2	1 vs 3	2 vs 3
*n*	20	23	16				
Age (years)	33±9	40±13	49±12	<0.001	0.06	<0.001	0.04
BMI (kg/m^2^)	22.7±1.2	30.6±2.3	31.8±4.3	<0.001	<0.001	<0.001	0.32
Weight (kg)	77.4±5.6	103.7±10.9	100.5±15.7	<0.001	<0.001	<0.001	0.49
Total body fat (kg)	12.5±4.2	26.6±6.5	28.9±8.4	<0.001	<0.001	<0.001	0.38
Fat percentage (%)	16.4±5.2	25.9±4.8	28.8±4.6	<0.001	<0.001	<0.001	0.06
UB fat (kg)	7.4±2.5	17.1±4.2	20.1±5.9	<0.001	<0.001	<0.001	0.09
UB SAT (kg)	6.0±1.9	13.6±3.4	15.8±5.3	<0.001	<0.001	<0.001	0.15
Abdominal fat (kg)	3.0±1.3	7.8±2.3	9.3±2.9	<0.001	<0.001	<0.001	0.11
Abdominal SAT (kg)	1.6±0.7	4.3±1.5	5.0±2.1	<0.001	<0.001	<0.001	0.32
VAT (kg)	1.3±0.6	3.5±1.1	4.3±1.1	<0.001	<0.001	<0.001	0.04
VAT/SAT	0.83±0.24	0.86±0.35	0.96±0.33	0.48	0.76	0.22	0.40
LB fat (kg)	5.2±1.9	9.6±2.7	8.8±2.7	<0.001	<0.001	<0.001	0.40
sCD163 (mg/l)	1.4 (0.8–1.7)	1.5 (0.5–2.8)	2.1 (0.7–3.0)	0.009	0.46	0.005	0.014
HbA1c (%)	5.3±0.3	5.3±0.3	6.6±0.9	<0.001	0.54	<0.001	<0.001
ALAT (U/l)	22 (11–36)	38 (18–96)	53 (22–106)	0.001	0.001	0.001	0.11
Glucose (mmol/l)	5.0 (4.2–5.8)	5.3 (4.3–6.1)	6.9 (5.5–13.8)	<0.001	0.10	<0.001	<0.001
Insulin (pmol/l)	18 (6–33)	40 (17–99)	64 (22–137)	<0.001	<0.001	<0.001	0.004
HOMA-IR	4.0 (1.1–8.0)	9.0 (3.6–23.2)	21.4 (5.5–57.6)	<0.001	<0.001	<0.001	<0.001
Triglyceride (mmol/l)	0.9 (0.5–1.8)	1.2 (0.6–2.3)	1.7 (1.2–3.8)	<0.001	0.006	<0.001	0.005
Total cholesterol (mmol/l)	4.6 (3.3–6.2)	5.1 (3.6–6.3)	4.5 (3.8–6.6)	0.07	0.04	0.10	0.09
HDL-cholesterol (mmol/l)	1.3 (0.8–1.8)	1.2 (0.7–1.8)	1.1 (0.8–1.8)	0.06	0.13	0.03	0.18
LDL-cholesterol (mmol/l)	2.8 (1.3–4.3)	3.2 (1.3–4.2)	2.5 (1.9–4.7)	0.06	0.06	0.63	0.04

T2DM, type 2 diabetes mellitus; UB, upper-body; SAT, subcutaneous adipose tissue; VAT, visceral adipose tissue; VAT/SAT, VAT/abdominal SAT; LB, lower-body; sCD163, soluble CD163; ALAT, alanine transaminase.

**Table 2:** Relationship between adiposity parameters and sCD163 in linear regression analyses. The association between adiposity parameters and ALAT vs sCD163 was investigated using a multiple linear regression model on a log–log scale. Multivariate analysis model 1 is adjusted for BMI and age (except for BMI, which is adjusted for age only).

**Group**	**Univariate analysis**			**Multivariate analysis model 1**	
*β*	*R*	*P*	*β*	*P*
Log BMI (kg/m^2^)					
1	−0.18	−0.04	0.90	−0.28	0.86
2	1.97	0.39	0.04	2.17	0.04
3	−0.84	−0.27	0.22	−0.68	0.36
Log total body fat (kg)					
1	−0.05	−0.08	0.80	0.03	0.91
2	0.70	0.52	0.007	0.92	0.007
3	−0.20	−0.13	0.52	0.27	0.63
Log fat percentage (%)					
1	−0.07	−0.10	0.75	0.02	0.95
2	1.08	0.57	0.003	1.33	0.002
3	−0.03	−0.01	0.95	0.65	0.41
Log UB fat (kg)					
1	−0.04	−0.07	0.84	0.08	0.76
2	0.71	0.53	0.006	0.98	0.004
3	−0.15	−0.10	0.63	0.40	0.45
Log UB SAT (kg)					
1	−0.04	−0.06	0.86	−0.02	0.92
2	0.75	0.54	0.005	0.87	0.01
3	−0.30	−0.23	0.27	−0.09	0.84
Log abdominal fat (kg)					
1	−0.06	−0.12	0.61	0.06	0.74
2	0.55	0.47	0.02	0.79	0.005
3	0.03	0.02	0.93	0.62	0.21
Log abdominal SAT (kg)					
1	−0.07	−0.13	0.70	−0.14	0.47
2	0.43	0.43	0.03	0.38	0.11
3	−0.27	−0.26	0.22	−0.43	0.25
Log VAT (kg)					
1	−0.04	−0.10	0.75	0.08	0.61
2	0.32	0.36	0.05	0.50	0.009
3	0.84	0.53	0.01	1.21	0.002
Log VAT/SAT					
1	0.01	0.01	0.97	−0.05	0.85
2	−0.02	−0.02	0.92	0.01	0.97
3	0.79	0.68	0.002	1.15	0.0001
Log LB fat (kg)					
1	−0.05	−0.09	0.79	−0.08	0.69
2	0.49	0.43	0.03	0.51	0.05
3	−0.27	−0.19	0.37	−0.30	0.53
Log ALAT					
1	0.24	0.32	0.25	0.22	0.33
2	0.50	0.60	0.003	0.50	0.0004
3	0.58	0.74	<0.0001	0.58	0.0001

*β*, regression coefficient; *R*, correlation coefficient. Group 1, normal-weight healthy men; group 2, overweight/obese non-diabetic men; group 3, overweight/obese men with type 2 diabetes mellitus. Test for identical regression coefficients for the three groups in the multivariate analysis: BMI, *P*=0.061; total body fat, *P*=0.033; fat percentage, *P*=0.014; UB fat, *P*=0.036; UB SAT, *P*=0.019; abdominal fat, *P*=0.045; abdominal SAT, *P*=0.036; VAT, *P*=0.007; VAT/SAT, *P*=0.002; LB fat, *P*=0.073; ALAT, *P*=0.384.

**Table 3:** Relationship between adiposity parameters and sCD163 in linear regression analyses. The association between adiposity parameters and sCD163 was investigated using a multiple linear regression model on a log–log scale. Multivariate analysis model 2 was used, including UB SAT, VAT, and LB fat and adjusted for BMI and age.

**Group**	**Multivariate analysis model 2**	
*β*	*P*
Log UB SAT (kg)		
1	0.20	0.72
2	0.90	0.11
3	−0.27	0.61
Log VAT (kg)		
1	−0.11	0.75
2	−0.06	0.80
3	1.69	<0.0001
Log LB fat (kg)		
1	−0.05	0.90
2	−0.08	0.82
3	−0.93	0.13

*β*, regression coefficient. Group 1, normal-weight healthy men; group 2, overweight/obese non-diabetic men; group 3, overweight/obese men with type 2 diabetes mellitus.
